# Development of 3D Printed pNIPAM-Chitosan Scaffolds for Dentoalveolar Tissue Engineering

**DOI:** 10.3390/gels10020140

**Published:** 2024-02-12

**Authors:** Mehdi Salar Amoli, Resmi Anand, Mostafa EzEldeen, Liesbet Geris, Reinhilde Jacobs, Veerle Bloemen

**Affiliations:** 1 Surface and Interface Engineered Materials (SIEM), Campus Group T, KU Leuven, Andreas Vesaliusstraat 13, 3000 Leuven, Belgium; mehdi.salaramoli@kuleuven.be (M.S.A.); resmi.anand@list.lu (R.A.); 2 OMFS IMPATH Research Group, Faculty of Medicine, Department of Imaging and Pathology, KU Leuven and Oral and Maxillofacial Surgery, University Hospitals Leuven, Kapucijnenvoer 33, 3000 Leuven, Belgium; mostafa.ezeldeen@kuleuven.be (M.E.); reinhilde.jacobs@kuleuven.be (R.J.); 3 Prometheus, Division of Skeletal Tissue Engineering Leuven, KU Leuven, 3000 Leuven, Belgium; liesbet.geris@kuleuven.be; 4 Department of Oral Health Sciences, KU Leuven and Paediatric Dentistry and Special Dental Care, University Hospitals Leuven, Kapucijnenvoer 33, 3000 Leuven, Belgium; 5 Biomechanics Research Unit, GIGA-R In Silico Medicine, University of Liège, Quartier Hôpital, Avenue de l’Hôpital 11, 4000 Liège, Belgium; 6 Biomechanics Section, KU Leuven, Celestijnenlaan 300C (2419), 3000 Leuven, Belgium; 7 Department of Dental Medicine, Karolinska Institutet, 171 77 Stockholm, Sweden

**Keywords:** pNIPAM, chitosan, degradability, 3D printing, dental pulp stem cells

## Abstract

While available treatments have addressed a variety of complications in the dentoalveolar region, associated challenges have resulted in exploration of tissue engineering techniques. Often, scaffold biomaterials with specific properties are required for such strategies to be successful, development of which is an active area of research. This study focuses on the development of a copolymer of poly (N-isopropylacrylamide) (pNIPAM) and chitosan, used for 3D printing of scaffolds for dentoalveolar regeneration. The synthesized material was characterized by Fourier transform infrared spectroscopy, and the possibility of printing was evaluated through various printability tests. The rate of degradation and swelling was analyzed through gravimetry, and surface morphology was characterized by scanning electron microscopy. Viability of dental pulp stem cells seeded on the scaffolds was evaluated by live/dead analysis and DNA quantification. The results demonstrated successful copolymerization, and three formulations among various synthesized formulations were successfully 3D printed. Up to 35% degradability was confirmed within 7 days, and a maximum swelling of approximately 1200% was achieved. Furthermore, initial assessment of cell viability demonstrated biocompatibility of the developed scaffolds. While further studies are required to achieve the tissue engineering goals, the present results tend to indicate that the proposed hydrogel might be a valid candidate for scaffold fabrication serving dentoalveolar tissue engineering through 3D printing.

## 1. Introduction

Dentoalveolar complications such as congenital abnormalities, tooth loss, periodontitis, or alveolar bone loss, affect a large portion of the global population, negatively affecting their quality of life. A wide range of available treatments have been used to address these complications, such as the use of dental implants or tooth autotransplantation [1,2]. While such advanced treatments have significantly enhanced the ability to treat a wide range of conditions, they often suffer from shortcomings. Challenges such as the inability to place implants in pediatric patients, the requirement for donor tissue in techniques such as autotransplantation, or the reliance on dentist abilities in treatment of periodontitis have resulted in the exploration of regenerative strategies in this region, where the natural ability of the tissues for regeneration is limited [3,4]. Among these strategies, tissue engineering approaches using cells combined with biomaterial-based scaffolds appear to be advantageous by providing a favorable environment for cell attachment and growth, as well as tissue regeneration [5,6,7]. In this regard, several aspects of the scaffolds, including the materials used for scaffold development and the fabrication strategy need to be considered. The dentoalveolar region is a heterogeneous tissue structure composed of complex geometries, housing several tissues such as dental pulp, dentin, enamel, periodontal ligament, and alveolar bone with a wide range of physical, mechanical and biological properties [5,8]. Hence, it demands scaffold fabrication methods capable of addressing such structural heterogeneity. Several traditional fabrication strategies have been suggested to develop scaffolds for tissue engineering in this region. These include solvent casting [9], multilayer hydrogel freeze drying [10], electrospinning [11], among others. However, such strategies often fail in recapitulating the complex geometrical complexity of the dentoalveolar region. Recently, 3D printing has been proposed as a potential fabrication technology capable of forming scaffolds with pre-determined geometries for different tissue engineering applications [12]. Capable of replicating designs based on patient imaging data or 3D scans, coupled with a layer by layer fabrication process, 3D printing enables development of geometries difficult to recreate using traditional approaches [13]. Consequently, different 3D printing and bioprinting strategies have been applied in the field of dentoalveolar tissue engineering, including extrusion-based, inkjet-based, and light-based strategies [14].

Among these, extrusion based strategies, based on formation of a continuous filament of scaffold material has gained attention due to the ease of creating 3D constructs, its versatility in terms of material properties, and low cost [14]. However, the development of materials which are compatible with the technology, and which possess the desired properties to be used in tissue engineering, such as biocompatibility or biodegradability, are still an important area of research in the field [15]. A range of material classes have been used for 3D printing of tissue engineering scaffolds, including thermoplastic polymers [16], bioceramics [17], and hydrogels [12]. Among these, hydrogels of both natural and synthetic origin are favorable for tissue engineering applications, because of their high water content, which resembles the natural extracellular matrix (ECM), and allows for cell survival and proliferation [18]. Furthermore, hydrogels often possess physical and mechanical properties closer to natural soft tissues and hence, are more suitable for tissue engineering strategies aimed at such structures [19]. Aside from these, physical and mechanical properties of this class of materials, such as their water content or their degradability can be tuned to range from days to months, making them suitable for a variety of applications [20].

Among the wide range of materials used for the development of tissue engineering hydrogels, chitosan, a polysaccharide produced from deacetylation of chitin, is one of the most interesting and widely investigated options. This is due to its high biocompatibility, biodegradability, anti-inflammatory properties, and antimicrobial activity [21]. However, similar to other natural polymers, it suffers from low mechanical properties, and a limited control over its physicomechanical characteristics. Consequently, several approaches such as crosslinking or copolymerization with other polymers have been suggested to enhance its properties [22,23]. For example, a composite of chitosan with alginate and hydroxyapatite has been demonstrated as useful in tissue engineering of bone tissue, enhancing the mechanical properties of chitosan and maintaining viability of MC3T3-E1 cells [24]. Similarly, chitosan—calcium phosphate composites have been shown to support dental pulp stem cell viability, proving useful as dental pulp capping agents.

Among different approaches for modification of chitosan, copolymerization with poly n-isopropylacrylamide (pNIPAM) is known to enhance properties of chitosan, in addition to conferring additional properties such as thermosensitivity [25,26]. pNIPAM is a synthetic biocompatible polymer used in a variety of biomedical applications such as cell sheet engineering [27] or drug delivery [28]. Furthermore, being a synthetic polymer, pNIPAM offers significant opportunity for tuning different properties of hydrogels such as their mechanical properties, enhancing the possibility of application in 3D printing. This has been shown by copolymerization of pNIPAM and polyethylene glycol (PEG), used for encapsulation of mesenchymal stem cells aimed at tissue engineering of cartilage tissue [29]. Similarly, pNIPAM-hydroxyapatite scaffolds have been used for bone tissue engineering, maintaining the viability of MG63 cells and used for release of antibiotics [30]. Demonstrating suitability of pNIPAM combination with natural polymers for 3D printing applications, it has been shown that hydrogels of pNIPAM, alginate and cellulose are useful for 3D printing of scaffolds capable of maintaining osteogenic ability of pre-osteoblastic cells cultured on the scaffolds [31].

Being one of the most important aspects of tissue engineering, utilization of pNIPAM may cause some concerns regarding its biodegradability. This is caused by lack of biodegradability of pNIPAM. However, its copolymerization with a natural polymer is known to result in degradable biomaterials as the natural component forms hydrogen bonds resulting in degradation of the polymer structure. This takes place due to the degradation of the natural component resulting in release of conjugated pNIPAM side chains [32]. Still, development of a chemically crosslinked pNIPAM-chitosan copolymer and its use as tissue engineering scaffolds or in 3D printing strategies has not been previously reported.

Consequently, this study hypothesizes that copolymerization of pNIPAM and chitosan through a sol-gel synthesis in the presence of a crosslinker, methylene-bis-acrylamide (MBA), could potentially result in 3D printable hydrogels which could be freeze dried and used as tissue engineering scaffolds capable of supporting dental pulp stem cells (DPSCs) viability and proliferation.

## 2. Results and Discussion

### 2.1. Sol-Gel Synthesis of pNIPAM-Chitosan Copolymer

**Scheme 1:**
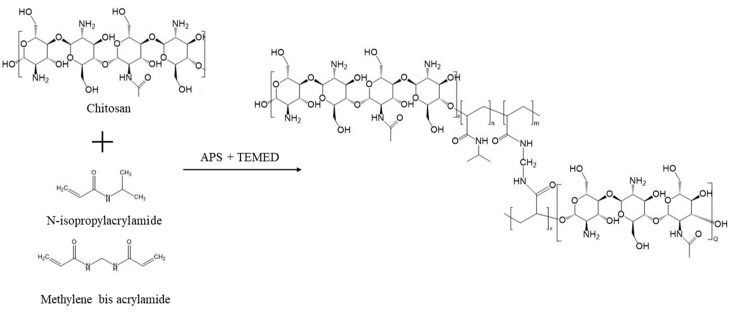
Schematic representation of the proposed reaction for synthesis of pNIPAM-chitosan copolymer.

To synthesize hydrogels of pNIPAM-chitosan copolymer, different components, including chitosan, NIPAM and MBA were dissolved in 1% (*w*/*v*) acetic acid. Addition of the radical initiator, APS, was followed by incorporation of TEMED, resulting in decomposition of APS through a redox reaction [33], making radicals available for the initiation of the polymerization. Following addition of TEMED, and due to the presence of MBA, the hydrogel backbone rapidly started to form, as demonstrated in Scheme 1. Among the nine different combinations of constituents, three combinations, namely samples with a chitosan:NIPAM ratio of 1:1, plus the sample with a chitosan:NIPAM ratio of 1:2 and a crosslinker concentration of 0.5 mol%, did not form a gel 24 h after addition of TEMED. The other five formulations resulted in a crosslinked gel within 1 h of TEMED addition.

### 2.2. Fourier Transform Infrared Spectroscopy (FTIR)

**Figure 1:**
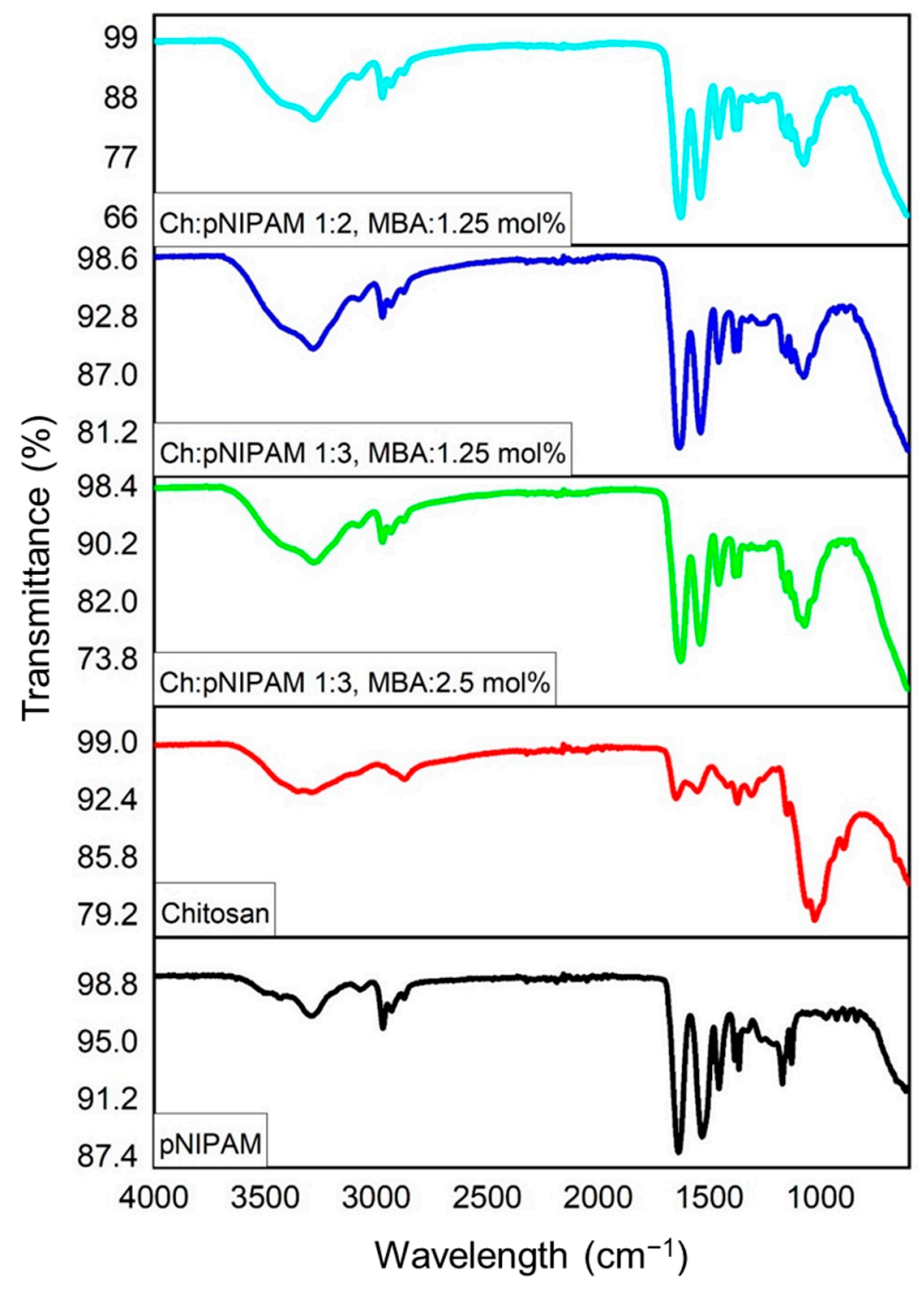
FTIR characterization of pNIPAM, chitosan and the copolymer with different ratios of constituents.

Fourier transform infrared spectroscopy (FTIR) was used to characterize the synthesized copolymer. Figure 1 demonstrates the peaks corresponding to different functional groups of chitosan, pNIPAM and chitosan-pNIPAM copolymer. As demonstrated, the peak ranging from 3100 to 3500 cm^−1^ corresponds to an OH/NH stretching vibration and it is intensified in the copolymer samples. Furthermore, the peak at 2866 cm^−1^ is intensified in the copolymer, signaling the crosslinking of the pNIPAM chains. In addition to these, the presence of peaks attributed to amide I and amide II of pNIPAM at 1534 and 1636 cm^−1^, along with the ether peak at 1077 cm^−1^ confirm the success of copolymerization [34,35,36,37].

### 2.3. Evaluation of Printability

**Figure 2:**
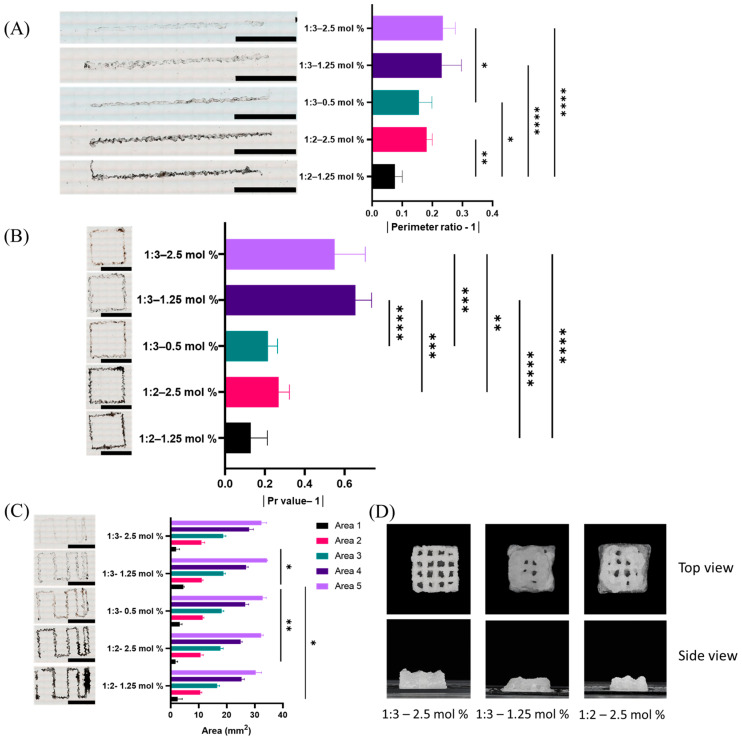
(**A**) evaluation of print fidelity by measurement of perimeter ratio (**B**) Evaluation of print fidelity by calculating the pr value (**C**) evaluation of print resolution by measuring the area between the lines and (**D**) printability in 3D. Scale bar 10 mm. *: *p* ≤ 0.05 **: *p* ≤ 0.01 ***: *p* ≤ 0.001 ****: *p* ≤ 0.0001.

The possibility of producing scaffolds of the synthesized hydrogel through 3D printing was evaluated by printing the synthesized gels on an extrusion based bioprinter. As demonstrated in Figure 2A, the lines printed using different hydrogel formulations result in a range of fidelities, with the most accurate ones being the sample synthesized using a chitosan:NIPAM ratio of 1:2 and an MBA concentration of 1.25 mol%, followed by 1:3, 0.5 mol% and 1:2, 2.5 mol%. In terms of resolution, considered to be the smallest possible distance between two distinguishable lines, samples with a material ratio of 1:3 crosslinked with 1.25 mol% were the best options, as shown in Figure 2C. In terms of pr value, best results were obtained from samples synthesized with a chitosan:NIPAM ratio of 1:2, and 1.25 mol% crosslinker, along with 1:3, and 0.5 mol% crosslinker, as demonstrated in Figure 2B. However, printing in three dimensions was not possible with samples of 1:2, and 1.25 mol% and 1:3, and 0.5 mol%. As shown in Figure 2D, the best shape fidelity in 3D was obtained from samples with a material ratio of 1:3 and a crosslinker concentration of 2.5 mol%.

### 2.4. Evaluation of Degradation and Swelling

**Figure 3:**
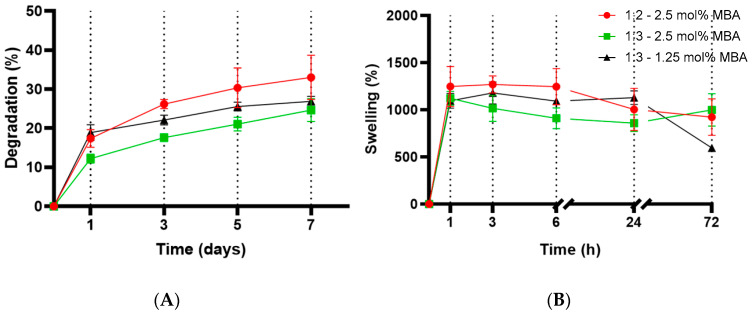
(**A**) Rate of scaffold hydrolytic degradation in water at 37 °C (**B**) Rate of scaffold swelling at 37 °C.

Considering the importance of scaffold degradation in tissue engineering strategies, the hydrolytic degradation rate of produced scaffolds in 37 °C water was measured through gravimetry. As demonstrated in Figure 3A, degradation after 7 days ranges from approximately 25% to 35%, with the formulation synthesized with a ratio of 1:2 and an MBA concentration of 2.5 mol% having a significantly higher degradation compared to the other two formulations (*p* < 0.05). Furthermore, the 1:3, 1.25 mol% formulation and the 1:3, 2.5 mol% had a similar rate of degradation after one week.

Additionally, the water content of the hydrogels can have a major impact on success of tissue engineering strategies. As presented in Figure 3B, the rate of swelling for all three hydrogel formulations was found to be approximately twelve times following one hour of immersion in deionized water. However, no significant differences among different samples were observed after one hour swelling. Furthermore, all the swelling took place in the first one hour of immersion, as no increase in the weight was observed in subsequent timepoints. The rate presented in Figure 3B, seems to reduce after 3 days of immersion in water.

### 2.5. Evaluation of Surface Morphology 

**Figure 4:**
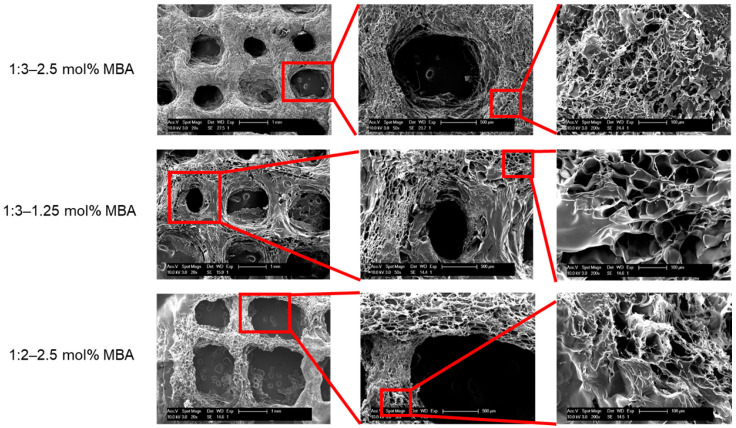
Surface morphology of the scaffolds obtained through SEM.

The surface morphology of the 3D-printed, freeze-dried scaffolds was evaluated through scanning electron microscopy (SEM). As shown in Figure 4, all three formulations show a high degree of porosity. Among these the average pore size of samples synthesized with a ratio of 1:3 and a 1.25 mol% crosslinker was significantly larger than other samples, being on average 6.3 × 10^3^ ± 176.6 µm^2^, while this average was 3.7 × 10^3^ ± 75.1 µm^2^ for samples with a material ratio of 1:2 and MBA concentration of 2.5 mol%, and 1.2 × 10^3^ ± 118.1 µm^2^ for samples of 1:3 material ratio and 2.5 mol% crosslinker.

### 2.6. Cell Viability

**Figure 5:**
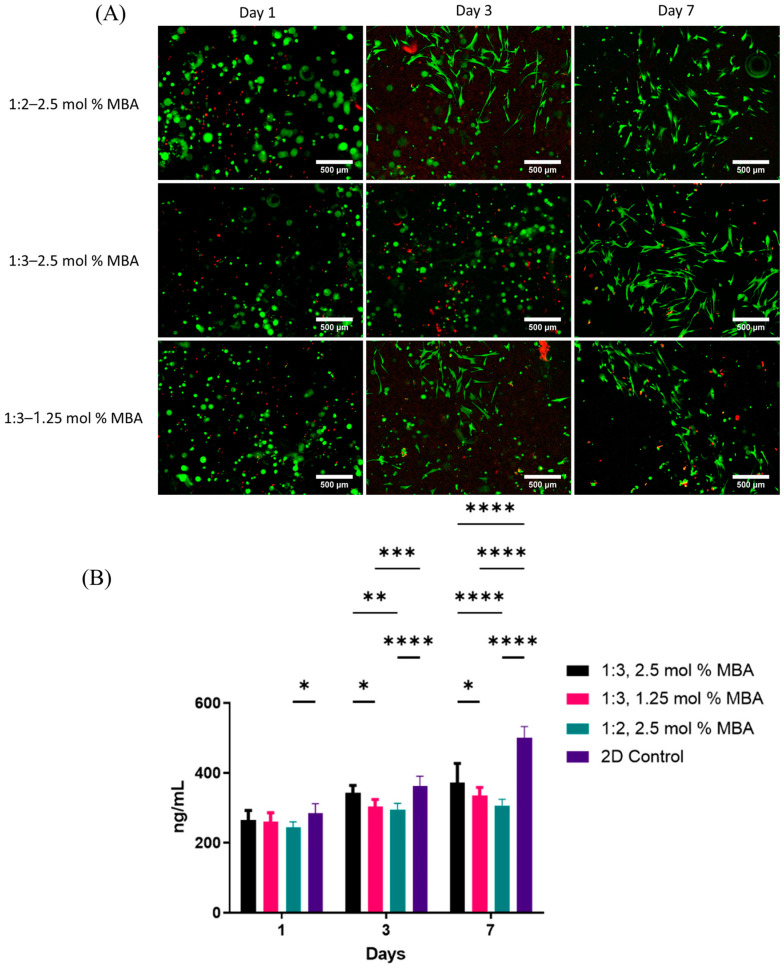
(**A**) Live/dead viability assay of DPSCs seeded on the scaffolds and (**B**) DNA quantification of DPSCs. *: *p* ≤ 0.05 **: *p* ≤ 0.01 ***: *p* ≤ 0.001 ****: *p* ≤ 0.0001.

To further evaluate the suitability of the designed scaffolds for tissue engineering applications and assess any potential cytotoxicity, dental pulp stem cells (DPSCs) were seeded and cultured on the scaffolds. The results of the live/dead viability assay demonstrated in Figure 5A show a much larger number of living cells compared to the dead cells, and no major differences can be observed among the three different hydrogel samples.

Additionally, to assess the cell viability in a quantitative manner, DNA quantification was performed on cells cultured on pNIPAM-chitosan scaffolds. The results, presented in Figure 5B, demonstrate capability of the scaffolds to support cell survival.

### 2.7. Further Discussion

In this study, hydrogels of pNIPAM-chitosan copolymer were synthesized through a one-step sol-gel polymerization reaction. Following the APS/TEMED reaction resulting in decomposition of APS, it can be hypothesized that the sulfate anion radicals (${S_{2}O}_{8}^{2-}$), produced by decomposition of APS, react with hydroxyl groups of chitosan, producing alkoxy radicals which then initiate copolymerization of NIPAM [37], as demonstrated in Scheme 1. Furthermore, presence of methylene-bis-acrylamide, results in the formation of crosslinks among polymer chains, leading to the development of hydrogels as the sol-gel reaction proceeds. The hypothesis for the reaction was confirmed using FTIR, where the shifting of the peak at 3317 to 3423 cm^−1^ is reported to confirm reaction on the hydroxyl groups [38], in addition to the presence of the specific peaks correlated to both materials.

Following the synthesis, 3D printing of these materials was performed to develop scaffolds with a potential use in tissue engineering applications. Consequently, 3D printing of the developed hydrogels was analyzed. While it was possible to print all synthesized formulations using extrusion printing, the quality of the prints was significantly different in 2D. Moreover, only three formulations were printable in 3D. As it appears that a higher crosslinking degree, resulting from both an increased amount of MBA, and an increased amount of NIPAM presenting a higher number of functional groups for crosslinking, results in an increased stability of the constructs enabling printing in 3D. As a result, considering the importance of printing in three dimensions for fabrication of tissue engineering scaffolds, formulations incapable of forming scaffolds in 3D were eliminated from further studies.

Being a synthetic polymer, pNIPAM alone is generally not considered as a degradable material [32], posing a challenge for tissue engineering applications using this material. However, copolymerization with natural polymers has been suggested as a method to induce biodegradability [39,40]. The efficiency of this method was confirmed through the results of this study, and the degradation behavior followed an expected trend, where a lower crosslinker concentration increased the rate of degradation [41]. Additionally, the rate of degradation is comparable to chitosan-based biomaterials, demonstrating the chitosan chains to be responsible for degradability of the copolymer [42].

Furthermore, the water content of the hydrogels was evaluated through a swelling study, where a swelling in the range of 1200% was observed. This swelling rate is lower than the reported swelling of pNIPAM at 20 °C [43], which could be attributed to the hydrophobic behavior of pNIPAM chains at 37 °C, however, it is well within the range observed in other chitosan-based scaffolds [44]. The slight reduction observed in the swelling rate after 72 h can be attributed to the hydrolytic degradation of the hydrogels, affecting the swelling measurements which are also based on the measurement of the weight [45]. Additionally, all formulations resulted in a high porosity with pore sizes ranging from 1.2 × 10^3^ to 6.3 × 10^3^ µm^2^, which is of great importance for transport of nutrients and waste to and from the cells, in addition to facilitating tissue ingrowth following implantation [46]. Among the samples in this study, it was observed that a lower rate of crosslinking resulted in larger pores as they are composed of a less tightly held network. Still, the pore size observed is much larger than the reported pore size of pure pNIPAM, being approximately 3.5 μm in diameter [47], and it is much closer to reported pore size of chitosan-based materials [48]. This is likely due to the significant impact of chitosan on the increased hydrophilicity of the copolymer, resulting in larger pore sizes in aqueous environment [49].

Following the physical characterization of the produced scaffolds, their ability to support viability of dental pulp stem cells (DPSCs) was evaluated. A qualitative analysis of the live/dead results would suggest that the material is suitable for maintaining the cell viability following a 7-day culture in medium with 1% FBS. This is consistent with previously reported observations showing a lack of toxicity by pNIPAM-based films [50]. Quantitatively, the DNA quantification results presented in Figure 5B, demonstrate the capability of the scaffolds to support cell survival. However, the rate of proliferation is lower than the control sample cultured in 2D. The higher rate in 2D culture is attributed to the culture plate coatings enhancing cell attachment. Additionally, the findings show that a larger number of cells are present on highly crosslinked scaffolds with a material ratio of 1:3, suggesting DPSCs prefer the increased mechanical properties. This behavior, and the effect of mechanical properties on behavior of DPSCs has been reported previously [51].

## 3. Conclusions

In this study, hydrogels based on a crosslinked copolymer of pNIPAM and chitosan crosslinked with MBA were developed through sol-gel synthesis. It was demonstrated for the first time that these hydrogels could be used for fabrication of scaffolds through 3D printing. Further characterization of the hydrogels demonstrated basic requirements for tissue engineering, including degradability, a high swelling rate and comparatively large pore sizes. In addition, DPSCs seeded on the scaffolds demonstrated viability of up to approximately 75% after 7 days of culture, demonstrating lack of any cytotoxicity caused by the copolymer. While further research is required to optimize the material properties, and evaluate the biological response of the cells, the results presented here suggest that the proposed material and fabrication method are suitable for the development of scaffolds. Further research using this novel biomaterial could include enhanced optimization of the material for printing, evaluation of rheological properties, along with in-depth biological evaluation of cell-based implants.

## 4. Materials and Methods

### 4.1. Materials

N-isopropylacrylamide (NIPAM) (97% purity), methylene-bis-acrylamide (MBA), chitosan from shrimp shell (molecular weight 190–310 kDa, deacetylation rate 75–85%), glacial acetic acid, ammonium persulfate (APS), and tetramethylethylenediamine (TEMED) were obtained from Sigma Aldrich (Overijse, Belgium). Calcein AM, ethidium homodimer, phosphate buffered saline (PBS), Gibco minimum essential alpha medium, gibco antibiotic-antimycotic, and Biowest fetal bovine serum (FBS) were obtained from ThermoFisher Scientific (Merelbeke, Belgium).

### 4.2. Methods

#### 4.2.1. Sol-Gel Synthesis of pNIPAM-Chitosan Copolymer

Chitosan was dissolved in 1% (*v*/*v*) glacial acetic acid in a round bottom flask at 60 °C overnight with a concentration of 25 mg/mL, and cooled down to room temperature afterwards. The chitosan solution was purged with nitrogen, and an appropriate amount of NIPAM, according to Table 1, was dissolved separately in 1.5 mL deionized water under nitrogen atmosphere. The NIPAM solution was added to the round bottom flask dropwise. Appropriate amounts of APS and MBA, according to Table 1, were dissolved in 1 mL water under nitrogen atmosphere, and added to the reaction chamber dropwise. The solution was allowed to stir for 30 min to ensure complete mixing, and 25 μL TEMED was added to start the reaction. The reaction was allowed to continue until the gel was formed. The gels were dialyzed against deionized water using dialysis tubing with a 14 kDa molecular weight cutoff to remove acetic acid and unreacted molecules for 3 days, with two water changes every day.

#### 4.2.2. Fourier Transform Infrared Spectroscopy (FTIR)

The FTIR-ATR spectra of the freeze-dried hydrogels was obtained using a spectrometer with the attenuated total reflection (ATR) (Vertex 70, Bruker, MA, USA), with a wavelength range of 600–4000 cm^−1^, a resolution of 2 cm^−1^ and an average of 32 scans.

#### 4.2.3. 3D Printability of the Scaffolds

The possibility of 3D printing the hydrogels was evaluated using an extrusion bioprinter (3DDiscovery, RegenHU, Villaz-Saint-Pierre, Switzerland). Gels were transferred to cartridges with a diameter of 7.29 mm, and several shapes for different analysis were printed using a 250 μm nozzle through a piston driven extrusion printing modality. To analyze one-dimensional shape fidelity, lines of 3 cm were printed, the perimeter of the lines was then measured and compared to the theoretical value. Additionally, to evaluate the two-dimensional accuracy of the print, squares were printed and a circularity factor was calculated as previously reported [17], where a value of closer to one represents a more accurate print. Furthermore, the resolution of the prints was evaluated by printing lines with different distances ranging from 1 mm to 10 mm and the area between the lines was measured as a signal of printing accuracy, and possibility of 3D printing was evaluated by printing cubes of 1 cm by 1 cm consisted of ten layers. Only samples capable of printing in 3D were selected for further tests.

#### 4.2.4. Evaluation of Degradation and Swelling

Samples for study of the hydrolytic degradation were prepared through 3D printing cubes of 10 mm by 10 mm by 5 mm made from different hydrogel formulations. Printed samples were then freeze dried (Alpha 1–2 LD plus, Martin Christ Freeze Dryers, Osterode, Germany), weighed and incubated in deionized water at 37 °C for 1, 3, 5 and 7 days. At each timepoint, samples were removed, freeze dried and weighed. The percentage of hydrolytic degradation was calculated through Equation (1), where *W*_0_ is the initial weight, and *W* is the weight after incubation.

(1) $$R_{d}=\frac{W_{0}-W}{W_{0}}\times 100$$

For swelling study, samples were prepared in the same way as samples for degradation study, except they were not freeze dried after incubation. Measurements for swelling were performed at 1, 3, 6, 24 and 72 h after incubation and swelling was calculated following Equation (2), where *W*_0_ is the initial weight and Wsw is the swollen weight.

(2) $$E_{sw}=\frac{W_{sw}-W_{0}}{W_{0}}\times 100$$

#### 4.2.5. Evaluation of Surface Morphology

To evaluate the surface morphology of the scaffolds, scaffolds of 10 mm by 10 mm by 5 mm with a grid infill and a distance of 2 mm between grids were printed, and freeze dried. The surface morphology of freeze-dried scaffolds were analyzed by coating the samples with 5 nm platinum/palladium (Q150T Plus, Quorum, Sussex, UK), followed by scanning electron microscopy (SEM) using a Philips scanning electron microscope XL30 at 10 KV.

#### 4.2.6. Cell Viability

Scaffolds of 0.7 mm by 0.7 mm and one layer, with a full infill were printed and freeze dried to assess cell viability on the scaffolds. Scaffolds were then sterilized under UV-C for 2 h. Dental pulp stem cells (DPSCs) were cultured according to previously described protocol [52]. DPSCs at passage 11 were seeded on the scaffolds with a density of 1 × 10^4^ cells/cm^2^. Scaffolds were then immersed in αMEM supplemented with 1% FBS and 1% antibiotics, and viability of seeded cells was evaluated at day 1, 3 and 7 following the seeding. For this, live/dead staining was used with Calcein AM and Ethidium homodimer (Live/Dead viability cytotoxicity kit, Invitrogen, Thermofisher Scientific) according to the manufacturer’s protocol. Fluorescent imaging was performed using an Olympus IX83 inverted microscope, and images were subjected to non-linear modifications removing the background signal.

DNA quantification was performed to quantitatively assess the cell viability and proliferation on the scaffolds. For this purpose, DPSC seeded scaffolds were prepared following the same protocol for live/dead assay. At each timepoint, medium covering the scaffolds was removed, 350 μL commercial RLT lysis buffer (Qiagen, Belgium) plus 1% (*v*/*v*) Β-mercapto-ethanol were added to the scaffolds, the samples were transferred to a microcentrifuge tube and vortexed for 1 min to prepare the lysate. Quant-iTTM dsDNA HS reagent was diluted in Quant-iTTM dsDNA HS buffer (Quant-iT dsDNA Assay Kit, Thermofisher Scientific) with a 1:200 ratio to make the working solution. Next, 5 μL of lysate was mixed with 195 μL of the working solution, and readings were performed using a Qubit fluorometer.

#### 4.2.7. Statistical Analysis

All experiments in different sections were performed in three independent replicates (*n* = 3). Analysis of Variance (ANOVA), followed by Tukey’s post-hoc test, was performed to assess differences using Graphpad Prism (version 8.0.0 for Windows, GraphPad Software, San Diego, CA, USA, www.graphpad.com (accessed on 1 January 2024)).

## Figures and Tables

**Table 1:** Different formulations used for sol-gel synthesis of hydrogels.

Chitosan/NIPAM Weightratio	APS (mol%)	MBA (mol%)	TEMED (μL)
1:1	4	0.5	25
1:2	4	0.5	25
1:3	4	0.5	25
1:1	4	1.25	25
1:2	4	1.25	25
1:3	4	1.25	25
1:1	4	2.45	25
1:2	4	2.45	25
1:3	4	2.45	25

## Data Availability

While synthesis of a copolymer of pNIPAM and chitosan has been reported before, in this study a novel approach by inclusion of methylene-bis-acrylamide was suggested, resulting in a one-step sol-gel synthesis of the biomaterial. Furthermore, it was demonstrated for the first time that this material could be used for development of tissue engineering scaffolds through 3D printing, ena-bling direct seeding of DPSCs on the hydrogel scaffold without causing cytotoxicity. This suggests this material as a novel candidate for further research on tissue engineering in the dentoalveolar region using advanced 3D printing strategies. All data and materials are available on request from the corresponding author. The data are not publicly available due to ongoing researches using a part of the data.
